# Intense Physical Exercise Induces an Anti-inflammatory Change in IgG N-Glycosylation Profile

**DOI:** 10.3389/fphys.2019.01522

**Published:** 2019-12-20

**Authors:** Marko Tijardović, Domagoj Marijančević, Daniel Bok, Domagoj Kifer, Gordan Lauc, Olga Gornik, Toma Keser

**Affiliations:** ^1^Faculty of Pharmacy and Biochemistry, University of Zagreb, Zagreb, Croatia; ^2^Endocrinology Laboratory, Department of Oncology and Nuclear Medicine, University Hospital Centre Sestre Milosrdnice, Zagreb, Croatia; ^3^Faculty of Kinesiology, University of Zagreb, Zagreb, Croatia; ^4^Genos Glycoscience Research Laboratory, Zagreb, Croatia

**Keywords:** IgG, glycans, N-glycome, inflammation, exercise, repeated sprint training

## Abstract

Exercise is known to improve many aspects of human health, including modulation of the immune system and inflammatory status. It is generally understood that exercise reduces inflammation, but there are missing links in terms of understanding the mechanisms as well as the differences between exercise modalities. N-glycosylation of immunoglobulin G (IgG) and total plasma proteins was previously shown to reflect changes in inflammatory pathways, which could provide valuable information to further clarify exercise effects. In order to further expand the understanding of the relationship between physical activity and inflammation, we examined the effect of intense exercise, in the form of repeated sprint training (RST), on IgG and total plasma proteins N-glycosylation in combination with traditionally used inflammation markers: C-reactive protein (CRP), interleukin 6 (IL-6), and leukocyte count. Twenty-nine male physical education students were separated into treatment (RST, *N* = 15) and control (*N* = 14) groups. The RST group completed a 6-week exercise protocol while the control group was instructed to refrain from organized physical activity for the duration of the study. Three blood samples were taken at different time points: prior to start of the training program, the final week of the exercise intervention (EXC), and at the end of the 4-week recovery period (REC). Following the end of the recovery period IgG N-glycosylation profiles showed anti-inflammatory changes in RST group compared to the control group, which manifested as a decrease in agalactosylated (*p* = 0.0473) and an increase in digalactosylated (*p* = 0.0473), and monosialylated (*p* = 0.0339) N-glycans. Plasma protein N-glycans didn’t change significantly, while traditional inflammatory markers also didn’t show significant change in inflammatory status. Observed results demonstrate the potential of intense physical exercise to reduce levels of systemic basal inflammation as well as the potential for IgG N-glycosylation to serve as a sensitive longitudinal systemic inflammation marker.

## Introduction

Exercise has long been known to improve many aspects of human health, having wide range of beneficial effects on musculoskeletal, metabolic and even cognitive functions. As a result, continuous physical exercise reduces the risk of cardiovascular diseases, diabetes, certain types of cancer, osteoporosis as well as depression (Kruk, 2007). Furthermore, many studies have shown that exercise reduces levels of basal inflammation which could be one of the mechanisms responsible for the observed health benefits (Beavers et al., 2010; Hamer et al., 2012; Pedersen, 2017).

During and after synthesis, most proteins undergo structural modification in which glycans, complex oligosaccharides, are attached to their backbone. This process is called glycosylation and blood plasma proteins are no exception to this mechanism. Most glycans present on plasma proteins are classified as N-glycans since they are attached by amide linkage to the nitrogen of the protein’s asparagine. In order for asparagine to be able to receive N-glycan, it has to be part of the Asn-X-Ser/Thr amino acid sequence, where X is any amino acid except proline. Glycosylation is a highly regulated process which is in different ways affected by almost any physiological and pathophysiological change in the organism. As a result, provoked changes in glycosylation are not random and they show repeatable patterns for different ways the homeostasis is being disrupted. Glycans regulate the cellular and humoral immune responses, including assembly of peptide-loaded major histocompatibility complexes (MHC) antigens, reorganization of T-cell receptor complexes, modulation of immune receptor clustering, endocytosis, receptor signaling, and immunoglobulin functions (Verhelst et al., 2019). The immune system contains different classes of glycan-binding proteins, including C-type lectins, galectins, and siglecs, which are expressed by immune cells and can be secreted. Glycan-binding proteins regulate leukocyte trafficking, pathogen recognition, immune cell activation, and immunosuppression (Verhelst et al., 2019). Many studies have found altered glycosylation patterns in chronic inflammatory and autoimmune diseases, such as rheumatoid arthritis (Parekh et al., 1985), inflammatory bowel disease (Trbojević Akmačić et al., 2015), systemic lupus erythematosus (Tomana et al., 1992), diabetes mellitus (Keser et al., 2017), cardiovascular disease risk and subclinical atherosclerosis (Menni et al., 2018), and many others, such as cancer and infections (Ohtsubo and Marth, 2006; Moremen et al., 2012; Pinho and Reis, 2015). Although less pronounced, it is even possible to associate lifestyle habits such as smoking with individual’s glycan profile (Knežević et al., 2010). These observations demonstrate the wide scope of glycan involvement in chronic diseases, indicating that changes in glycosylation could be used as diagnostic and prognostic markers for many different diseases as well as assessing the general health status of an individual.

Apart from being the most abundant antibody in our body, IgG is also one of the most studied glycoproteins. Each IgG molecule has two conserved glycosylation sites, one for each of its heavy chains at Asn-297 of the constant heavy (CH2) domain (Krapp et al., 2003). In addition to conserved IgG Fc glycans, ∼15–25% of serum IgG contains glycans within the variable domains (van de Bovenkamp et al., 2016). Different glycans attached to these glycosylation sites can alter IgG’s conformation, stability and half-life, but even more importantly, its affinity for Fc receptors. By changing this affinity, its effector functions are altered causing it to become more or less inflammatory (Jennewein and Alter, 2017). Glycans that lack terminal galactose activate complement and make IgG pro-inflammatory, while the addition of galactose decreases inflammatory potential of IgG (Malhotra et al., 1995; Karsten et al., 2012; Mihai and Nimmerjahn, 2013). Further extension of IgG glycans by the addition of sialic acid dramatically changes the physiological role of IgG, converting it from a pro-inflammatory into an anti-inflammatory agent. Terminal α2,6-sialylation of IgG glycans decreases the ability of IgG to bind to activating FcγRs and promotes recognition by the C-type lectin dendritic cell-specific intercellular adhesion molecule (ICAM)-grabbing non-integrin (DC-SIGN), which increases expression of inhibitory FcγRIIB and is anti-inflammatory (Anthony et al., 2011; Quast et al., 2015). However, these findings have not been confirmed in all studies (Boyd et al., 1995; Campbell et al., 2014; Issekutz et al., 2015). IgG galactosylation is strongly associated with aging and it decreases to less than 50% of its maximal value through lifetime (Yamada et al., 1997; Krištić et al., 2014). Only three IgG glycans are sufficient to explain up to 58% of variance in chronological age, significantly more than other markers of biological age like telomere lengths (Krištić et al., 2014). Furthermore, it is well-established that increased abundance of agalactosylated IgG glycans has been found in various diseases and states with an underlying inflammatory component: infectious diseases [hepatitis C infection (Mehta et al., 2008), HIV infection (Moore et al., 2005)], autoimmune diseases [rheumatoid arthritis (Parekh et al., 1985, 1988; Van Zeben et al., 1994; van de Geijn et al., 2009), juvenile chronic arthritis (Bond et al., 1997; Flögel et al., 1997), Crohn’s disease (Dubé et al., 1990; Trbojević Akmačić et al., 2015), ulcerative colitis (Dubé et al., 1990; Trbojević Akmačić et al., 2015), systemic lupus erythematosus (Vučković et al., 2015), myositis (Perdivara et al., 2011)], and cancer [prostate cancer (Kanoh et al., 2004), lung cancer (Kanoh et al., 2006), ovarian cancer (Saldova et al., 2007), gastric cancer (Ruhaak et al., 2015), breast cancer (Kawaguchi-Sakita et al., 2016)]. At the same time, increased levels of IgG digalactosylation and sialylation are acting in an anti-inflammatory manner (Gudelj et al., 2018). Therefore, IgG glycosylation pattern cannot be used as a stand-alone disease-specific biomarker and is of more value as a biomarker of general immune activation (de Jong et al., 2016) and an excellent marker of a person’s general health state (Gudelj et al., 2018).

Very little research has been done on the effects of exercise either on total plasma N-glycosylation or IgG N-glycosylation. Recently published study (Sarin et al., 2019) investigated a number of molecular pathways, including IgG N-glycosylation, explaining immunosuppression in individuals undergoing prolonged periods of intense training with low-energy availability. They observed IgG glycosylation status alteration toward pro-inflammatory activity. However, changes in the abundance of distinct glycan peaks were mostly observed after the weight loss period and the effect of the intense exercise alone could not be inferred (main aim of the study was to assess the effect of energy deprivation leading to substantial fat mass loss). Other relevant studies focused on relation of exercise and GlycA levels, a glycan biomarker of systemic inflammation, where decreasing levels of GlycA following exercise were observed (Bartlett et al., 2017; Barber et al., 2018). However, training interventions investigated in the aforementioned studies were all comprised of either moderate (40–55% VO2max) and/or high-intensity (65–80% VO2max) aerobic exercise (Bartlett et al., 2017; Barber et al., 2018). So far, there are no studies available in the literature in which inflammatory effects of anaerobic all-out exercises were investigated. Repeated sprint training (RST) is an increasingly popular training program comprised of short-duration maximal-effort sprints separated with up to 30-s recovery periods (Girard et al., 2011). This type of training induces great metabolic stress and may lead to acute attainment of VO2max (Buchheit and Laursen, 2013). Due to much greater intensity at which RST is executed in comparison to moderate and high-intensity aerobic training and due to much greater eccentric muscle contractions elicited while sprinting as opposed to running or jogging, leading to a greater level of subsequent muscle damage, this type of training may provide stimulus for greater anti-inflammatory adaptations. As short-duration high-intensity activities became very popular even among general population as a time-efficient strategy for health improvement (Batacan et al., 2017), it is of utmost importance to investigate its potential for inducing positive adaptations of the immune system. Therefore, we aimed to investigate the way 6-week RST affects IgG and total plasma N-glycan profiles in healthy individuals. In order to get more detailed insight into inflammatory activities, along with glycosylation, we also analyzed traditionally used inflammatory markers: C-reactive protein (CRP), interleukin 6 (IL-6) as well as leukocyte count. Additionally, as general population usually follows an undulating pattern of undergoing intensive training phases followed by periods of complete exercise cessation, which leads to detraining (Mujika and Padilla, 2000), we were also interested in residual effects of relatively short-volume RST on parameters of immune activity. Therefore, this longitudinal intervention study performed on 29 individuals out of which 14 served as controls, was designed with three assessment time-points: before and immediately after the penultimate training session of the 6-week training period and following 1-month detraining period.

## Materials and Methods

### Participants

Twenty-nine male physical education students volunteered to participate in the study. They were recruited for the study through oral announcements about commencement of the study which were delivered on several occasions at the beginning of their university classes. All participants were screened for cardiovascular diseases, muscle injuries or ongoing medical treatment using the Physical Activity Readiness Questionnaire (PAR-Q) before their inclusion into the experimental protocol. Basic inclusion criteria involved non-participation in any organized physical activity program or sport for 3 months before and throughout the study. Participants were, however, allowed to be physically active and exercise during practical classes they were obligated to undertake as their course requirement. As they were physical education students all participants were either former athletes or had been extensively trained in a certain sport, so they all were familiar with intermittent activities. Participants were instructed to refrain from alcohol and cigarette consumption as well as antioxidant supplementation throughout the study. For the purpose of the study the participants were randomly selected into either experimental (RST) or control group (Table 1). Before the commencement of the study, the experimental procedures and potential risks were fully explained to all participants and they all provided written informed consent. The study was designed in accordance with the Declaration of Helsinki and was approved by the Ethical Committee of the University of Zagreb Faculty of Pharmacy and Biochemistry.

**TABLE 1:** Descriptive data for participants in the experimental (RST) and control group shown as mean and standard deviation.

	RST *n* = 15	Control *n* = 14
Age (years)	20.0 ± 1.0	19.5 ± 0.7
Height (cm)	181.0 ± 4.4	180.2 ± 6.9
Weight (kg)	77.7 ± 6.0	77.8 ± 12.9
Body fat (%)	8.7 ± 3.0	9.8 ± 4.8

### Study Design

Intervention group underwent 6-week long exercise protocol which consisted of 18 progressively more intense exercise sessions. Main part of sessions included 2–3 sets with 6–10 repetitions of 20-m sprints, with departures every 25 s and 2-min inter-set passive recovery. Blood samples were taken simultaneously from all participants at three time points: before the training intervention protocol, immediately after the most intense, penultimate exercise session, and 1-month following the end of EXC protocol. For detailed protocol description and blood sampling information please refer to Supplementary Material.

### Laboratory Analysis of Traditional Inflammation Markers

C-reactive protein was measured in plasma down to very low concentrations on Beckman Coulter AU System (AU 2700 plus) with CRP Latex reagent (Highly sensitive application). In this procedure, decreasing intensity of transmitted light (increasing absorbance) through reaction solution reflects the formation of complexes during the immunological reaction between the subject’s plasma CRP and the rabbit anti-CRP-antibodies coated latex particles. The within-run precision was ≤5% CV or SD ≤ 0.02 mg/L and the total precision was ≤10% CV or SD ≤ 0.02 mg/L.

Interleukin-6 levels in plasma were measured using ultrasensitive sandwich ELISA assay as described in the manufacturer’s protocol (BioVendor Laboratory Medicine, Inc., Brno, Czechia). The standard curve ranged between 1.56 and 50 pg/mL IL-6. The assay recognizes both natural and recombinant human IL-6. To define the specificity of this ELISA, several proteins were tested for cross-reactivity. There was no cross-reactivity observed for any protein tested (IL-1a, IL-1b, IL-10, IL-12, IFNg, IL-4, TNFa, IL-8, and IL-13). The overall intra-assay coefficient of variation was calculated to be 4.4%.

A complete blood cell count was performed on ADVIA 2120 (Siemens Healthcare Diagnostics), immediately after sampling.

### IgG Isolation

Immunoglobulin G was isolated from plasma by affinity chromatography using 96-well protein G monolithic plates (BIA Separations, Ljubljana, Slovenia) as described previously (Pučić et al., 2011). Briefly, 100 μL of plasma was diluted 10× with PBS and then applied to the protein G plate and instantly washed. IgGs were eluted with 1 mL of 0.1 M formic acid (Merck, Darmstadt, Germany) and immediately neutralized with 1 M ammonium bicarbonate (Acros Organics, NJ, United States).

### IgG N-Glycans Release and Labeling

Dried IgG samples were resuspended and denatured by incubation with 30 μL SDS (1.33% wt/vol; Invitrogen, Carlsbad, CA, United States) at 65°C for 10 min. Subsequently, 10 μL of 4% Igepal-CA630 (Sigma-Aldrich, St. Louis, MO, United States) and 1.2 u PNGase F (Promega, Madison, WI, United States) in 10 μL 5 × PBS were added. The samples were incubated overnight at 37°C to allow release of N-glycans. The released N-glycans were labeled with 2-AB. The labeling mixture was freshly prepared by dissolving 2-AB (19.2 mg/ml; Sigma-Aldrich) and 2-picoline borane (44.8 mg/ml; Sigma-Aldrich) in a mixture of DMSO (Sigma-Aldrich) and glacial acetic acid (Merck, Darmstadt, Germany) (70:30, vol/vol). Labeling mixture (25 μL) was added to each N-glycan sample in the 96-well plate, which was then sealed using adhesive seal. Mixing was achieved by shaking for 10 min, followed by incubation at 65°C for 2 h. To each sample (75 μL), 700 μL of acetonitrile (ACN) (J. T. Baker, Phillipsburg, NJ, United States) was added. Free label and reducing agent were removed from the samples using HILIC solid-phase extraction (SPE). A GHP filter plate, 0.2 μm (Pall Corporation, Ann Arbor, MI, United States) was used as the stationary phase. All wells were prewashed using 1 × 200 μL of ethanol/water (70:30, vol/vol) and 1 × 200 μL water, followed by equilibration using 1 × 200 μL of ACN/water (96:4, vol/vol). Solvent was removed by the application of a vacuum using a vacuum manifold (Millipore Corporation, Billerica, MA, United States). The samples were loaded into the wells, which were subsequently washed five times using 200 μL of ACN/water (96:4, vol/vol). Glycans were eluted with 2 × 90 μL of water and combined eluates were stored at −20°C until usage.

### Total Plasma Proteins N-Glycans Release and Labeling

Total plasma proteins glycans were prepared in the same way as the IgG glycans. The only difference is that, instead with dried IgG eluate, the preparation began with 10 μL of blood plasma and with addition of 20 μL 2% SDS (w/v) (Invitrogen, Carlsbad, CA, United States) to each sample before the incubation at 65°C for 10 min.

### Hydrophilic Interaction Chromatography (HILIC)-UPLC Analysis of Labeled Glycans

Fluorescently labeled N-glycans were separated by HILIC on Waters Acquity ultra-performance liquid chromatography (UPLC) instrument (Milford, MA, United States) consisting of a quaternary solvent manager, sample manager, and a FLR fluorescence detector set with excitation and emission wavelengths of 250 and 428 nm, respectively. The instrument was under the control of Empower 2 software, build 2145 (Waters, Milford, MA, United States). Labeled N-glycans were separated on a Waters bridged ethylene hybrid (BEH) Glycan chromatography column, 100 × 2.1 mm i.d. for IgG glycans and 150 × 2.1 mm i.d. for total plasma proteins glycans, 1.7 μm BEH particles, with 100 mM ammonium formate, pH 4.4, as solvent A and acetonitrile as solvent B. The separation method used a linear gradient of 75–62% acetonitrile (v/v) at flow rate of 0.4 ml/min in a 25 min analytical run for IgG glycans and linear gradient of 70–53% acetonitrile (v/v) at flow rate of 0.561 ml/min in a 24.81 min analytical run for total plasma proteins glycans. The separation temperature was 60°C for IgG glycans and 25°C for total plasma proteins glycans. Samples were maintained at 10°C before injection. Data processing was performed using an automatic processing method with a traditional integration algorithm after which each chromatogram was manually corrected to maintain the same intervals of integration for all the samples. The chromatograms were all separated in the same manner into 24 peaks (Supplementary Figure S1) for IgG glycans and 39 peaks (Supplementary Figure S2) for total plasma proteins glycans and the amount of glycans in each peak was expressed as percentage of total integrated area. The system was calibrated using an external standard of hydrolyzed and 2-AB labeled glucose oligomers from which the retention times for the individual glycans were converted to glucose units. Glycan peaks were analyzed on the basis of their elution positions and measured in glucose units then compared to reference values in the “GlycoStore” database^1^ for structure assignment (Campbell et al., 2008; Abrahams et al., 2018). From directly measured glycan peaks, derived traits were calculated. These derived traits average particular glycosylation features (e.g., galactosylation, fucosylation, bisecting GlcNAc, sialylation) across different individual glycan structures, and consequently they are more closely related to individual enzymatic activities and underlying genetic polymorphisms. As derived traits represent sums of directly measured glycans, they were calculated using normalized glycan measurements (Supplementary Table S2).

### Statistical Analysis

From directly measured and normalized 24 glycan peaks for IgG and 39 glycan peaks for plasma proteins, derived traits were calculated following formulas in Supplementary Table S2. Statistical analysis was carried out using R programming language (version 3.5.1). In order to compare changes in derived traits as a result of exercise, before the statistical analysis was performed, each subjects result was expressed as an absolute change in individual glycan trait or level of inflammation marker, relative to the baseline measurement. Linear mixed regression model was used to perform the analysis, where change in individual measurement was set as dependent variable. Time point as well as group (treatment or control) were set as independent variables, with group variable being nested inside the time point variable. As a random intercept, subjects IDs were used to minimize effects of interindividual differences. To account for multiple testing, Benjamini–Hochberg correction was used to adjust the *p*-values.

In order to describe relations between glycan traits and inflammatory markers we calculated Kendall rank correlation coefficient and presented it with unadjusted significance as a correlation matrix.

## Results

To investigate the effects of intense physical training on proportions of N-glycans, we conducted a longitudinal intervention study and measured the samples in three time points: before, at the peak of training program intensity (EXC), and 1 month after the intervention (recovery period, REC). We measured IgG and total plasma N-glycomes from blood plasma samples for each time point and each subject. The samples were measured by HILIC-UPLC, which produces chromatogram with 24 glycan peaks for IgG N-glycome (Supplementary Figure S1) and 39 peaks for plasma N-glycome (Supplementary Figure S2). Derived glycan traits were calculated from directly measured glycan peaks (Supplementary Table S2). These derived traits represent common biologically meaningful features (agalactosylation, monogalactosylation, digalactosylation, sialylation, fucosylation, etc.) shared among several measured glycans (for more details see section “Materials and Methods”). Results were compared between treatment and control group to estimate the effect of RST protocol on measured parameters.

### Effect of Intense Exercise on IgG N-Glycosylation

**FIGURE 1:**
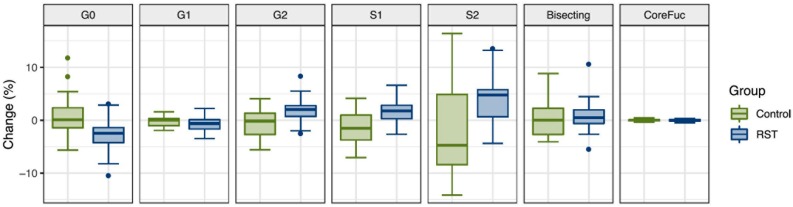
Box plots showing relative changes in the last time point (REC) compared to the baseline for each of derived IgG glycan traits.

After the analysis of IgG N-glycosylation, we compared results from EXC and REC time points to the baseline measurement for each of the seven following derived traits: agalactosylation, monogalactosylation, digalactosylation, monosialylation, disialylation, bisection, and core fucosylation. Calculated effect of exercise showed significant changes of IgG N-glycans in the last (REC) time point relative to the baseline, which includes decrease of agalactosylated N-glycans (−0.8080, *p* = 0.0473), and increase in digalactosylated and monosialylated N-glycans (0.9949, *p* = 0.0473 and 0.5270, *p* = 0.0339, respectively) (Table 2). Figure 1 shows changes of each IgG glycosylation derived trait from baseline to REC for each subject represented by boxplots. Interestingly, there were no significant changes in the EXC time point, although by looking at the data in Table 2 it is possible to see the trend in EXC toward the results observed in REC.

**TABLE 2:** Estimated effects of training intervention on levels of individual derived IgG N-glycan traits, and their respective unadjusted and adjusted *p*-values.

	Estimate EXC	*p*-value EXC	Adj. *p*-value EXC	Estimate REC	*p*-value REC	Adj. *p*-value REC
G0	−0.2009	0.5394	0.6152	**−0.8080**	**0.0190**	**0.0473**
G1	−0.1040	0.5388	0.6152	−0.1710	0.3149	0.4409
G2	0.3049	0.4554	0.6152	**0.9950**	**0.0203**	**0.0473**
S1	0.2688	0.1283	0.6152	**0.5270**	**0.0048**	**0.0339**
S2	0.1430	0.2206	0.6152	0.2465	0.0399	0.0698
Bisecting	0.0901	0.5807	0.6152	0.1095	0.5030	0.5868
CoreFuc	−0.0373	0.6152	0.6152	−0.0339	0.6479	0.6479

*The exercise effect coefficients are calculated relative to the baseline. Significant results (α = 0.05) are shown in bold.*

### Effect of Intense Exercise on Total Plasma Protein N-Glycosylation

**FIGURE 2:**
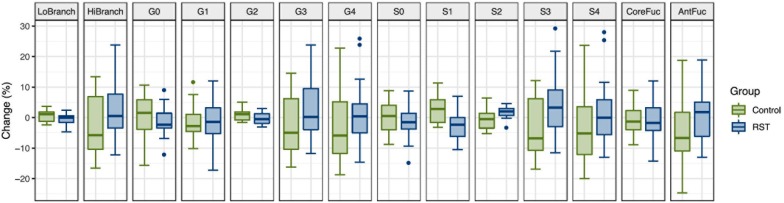
Box plots showing relative changes in the last time point (REC) compared to the baseline for each of derived total plasma protein glycan traits.

To determine whether intense exercise induces a more general change in glycosylation of multiple proteins, the effect of exercise on N-glycome of total plasma proteins was also investigated. Plasma samples were analyzed in the same way as IgG samples regarding time points and derived traits, while the only difference was presence of 14 instead of 7 calculated traits: low branching, high branching, agalactosylation, monogalactosylation, digalactosylation, trigalactosylation, tetragalactosylation, asialylation, monosialylation, disialylation, trisialylation, tetrasialylation, core fucosylation, and antennary fucosylation. The only significant effect of exercise on total plasma protein N-glycosylation was a decrease in monosialylated glycans in the third (REC) time point relative to the baseline (−1.2560, *p* = 0.0371) (Table 3). Figure 2 shows changes of each glycosylation derived trait from baseline to the end of the study represented by boxplots.

**TABLE 3:** Estimated effects of training intervention on levels of individual derived total plasma protein N-glycan traits, and their respective unadjusted and adjusted *p*-values.

	**Estimate EXC**	***p*-value EXC**	**Adj. *p*-value EXC**	**Estimate REC**	***p*-value REC**	**Adj. *p*-value REC**
LoBranch	−0.1896	0.7358	0.8905	−0.9541	0.0981	0.2746
HiBranch	0.1956	0.7270	0.8905	0.9672	0.0927	0.2746
G0	0.1120	0.6400	0.8905	−0.0980	0.6820	0.7956
G1	0.5400	0.2185	0.7646	0.0204	0.9623	0.9699
G2	−0.8416	0.1532	0.7646	−0.8765	0.1375	0.3209
G3	0.1965	0.6806	0.8905	0.8536	0.0821	0.2746
G4	−0.0009	0.9932	0.9932	0.1137	0.2924	0.4277
S0	0.7506	0.3763	0.8905	−0.4377	0.6041	0.7688
S1	−0.5685	0.1447	0.7646	**−1.2561**	**0.0027**	**0.0371**
S2	−0.3638	0.6154	0.8905	0.8110	0.2673	0.4277
S3	0.1806	0.6220	0.8905	0.7881	0.0387	0.2710
S4	0.0070	0.9374	0.9932	0.1079	0.2323	0.4277
CoreFuc	1.4520	0.1858	0.7646	0.0407	0.9699	0.9699
AntFuc	−0.0475	0.7632	0.8905	0.1632	0.3055	0.4277

*The exercise effect coefficients are calculated relative to the baseline. Significant results (α = 0.05) are shown in bold.*

### Effect of Intense Exercise on Traditional Inflammation Markers

C-reactive protein, IL-6 as well as leukocyte count have all been traditionally used to measure the extent of inflammation in patients, so we have also analyzed these markers to investigate the dynamics of change with exercise.

Opposite to the N-glycans, some inflammatory markers showed significant changes in the EXC time point while in the REC time point, after the recovery period, there were no changes compared to the baseline (Table 4). IL-6 as well as total leukocyte count was significantly increased in EXC (2.7837 pg/mL, *p* = 0.0006 and 1.4624 × 109/L, *p* = 0.0489, respectively). Neutrophil count also increased in EXC although the result was not statistically significant (1.2732 × 109/L, *p* = 0.0631).

**TABLE 4:** Estimated effects of training intervention on levels of inflammatory markers, and their respective unadjusted and adjusted *p*-values.

	**Estimate EXC**	***p*-value EXC**	**Adj. *p*-value EXC**	**Estimate REC**	***p*-value REC**	**Adj. *p*-value REC**
CRP (mg/L)	0.3074	0.5688	0.5688	0.4499	0.4059	0.8118
IL-6 (pg/mL)	**2.7837**	**0.0002**	**0.0006**	0.6835	0.2890	0.8118
Leu (10^9^/L)	**1.4624**	**0.0245**	**0.0489**	−0.1338	0.8287	0.8979
Neu (10^9^/L)	1.2732	0.0474	0.0632	0.0792	0.8979	0.8979

*The exercise effect coefficients are calculated relative to the baseline. Significant results (α = 0.05) are shown in bold.*

Furthermore, we investigated relationship between inflammation markers and derived glycan traits in the exercise group. For IgG N-glycans, we calculated the relationships of inflammatory markers with glycan traits related to galactose and sialic acid (Supplementary Figure S3A). Correlation coefficients were mostly weak and statistically insignificant, indicating there is no clear and strict relationship between derived IgG glycan traits and inflammatory markers.

Regarding total plasma proteins N-glycans we found moderate positive correlations with high branching glycans and antennary fucosylation and negative correlations with low branching glycans (Supplementary Figure S3B). Branching and antennary fucosylation are considered as inflammatory traits so the observed correlations were expected (Novokmet et al., 2015; Gudelj et al., 2016).

## Discussion

In this study we aimed to investigate the effects of long term intense physical training on IgG and total plasma N-glycan profiles in healthy individuals. We conducted a longitudinal intervention study and measured the samples in three time points: before, at the peak of training program intensity, and 1 month after the intervention.

Immunoglobulin G N-glycosylation is known to reflect inflammatory and anti-inflammatory processes occurring in the body. Our findings of decreased agalactosylated N-glycans and rise in digalactosylated and monosialylated N-glycans attached to IgG, 1 month after the end of the training protocol, suggest exercise indeed induced anti-inflammatory effects in the experimental group (Seeling et al., 2017; Gudelj et al., 2018). It is important to emphasize the fact that the observed changes in IgG glycans had the same anti-inflammatory trend in both measurements after the initial baseline measurement, despite leukocyte count being slightly elevated right after the end of EXC. This indicates that IgG N-glycans could be reflecting only general and long-term changes in inflammatory status of an individual, while not being greatly affected by acute variations which can be detected using traditional markers. Previous studies have also shown that IgG glycosylation has a great potential for monitoring and managing levels of inflammation in chronic disease patients, and other individuals with risk of inflammatory processes or even general population (Krištić et al., 2014; Novokmet et al., 2015; Menni et al., 2018). Our findings suggest that these changes in IgG glycosylation are sensitive enough to even detect subtle anti-inflammatory changes caused by an intense exercise and that IgG glycosylation could be a useful tool in assessment of new exercising methods for professional athletes. Moreover, increased levels of terminally galactosylated and sialylated IgG glycan structures in students who underwent RST indicates an improvement in their general health, since this trend of change in IgG glycosylation is associated with lower biological age and lower inflammatory status (Krištić et al., 2014; Gudelj et al., 2018). It would be interesting to investigate for how long these changes in IgG glycosylation would last, and if RST would have the same or larger effect on elder, overweight or sedentary individuals, who would largely benefit from anti-inflammatory effect of exercise. It is possible that IgG glycosylation could be used in assessing the anti-inflammatory effect of different exercise treatments in individuals with metabolic syndrome or similar conditions with underlying systemic inflammation. Pedersen and Saltin gathered evidence for therapeutic effect of exercise in 26 different chronic diseases (Pedersen and Saltin, 2015). IgG glycosylation could help to determine the best type and dose of exercise for each disease more precisely. Furthermore, this type of testing could prove to be beneficial for professional athletes in order to help them balance their training intensity and avoid development of overtraining syndrome (Carfagno and Hendrix, 2014).

In order to determine whether intense exercise induces a more general change in glycosylation of multiple proteins, we also investigated the effect of exercise on N-glycome of total plasma proteins. Decrease in total monosialylated N-glycans is the only glycosylation trait among 14 measured that was significantly different after the exercise in total plasma protein N-glycome. Since it is the only visible change, it is hard to distinguish the real cause and meaning of its change, because measured glycan levels in total plasma protein N-glycome are product of both composition of glycans attached to different proteins, as well as the relative abundance of those proteins.

Furthermore, to get more detailed insight into inflammatory activities, along with glycosylation, we also analyzed traditionally used inflammatory markers: CRP, interleukin 6 (IL-6) as well as leukocyte count. In accordance with literature, we found significant increase in plasma IL-6 following the most intense RST as a result of its release from the muscle tissue (Pedersen, 2017). IL-6 has both pro-inflammatory and anti-inflammatory properties (Scheller et al., 2011), but in context of exercise its anti-inflammatory effects dominate, and it is even considered as one of the drivers of beneficial anti-inflammatory effects of exercise (Pedersen, 2017). CRP is the most often used general marker of inflammation and in this study, we did not detect significant change in its concentration. Leukocyte count can also indicate presence of inflammation and here we detected elevated values after the most intense RST. This suggests some inflammatory activity, but the observed effect is likely arising from local acute muscle tissue inflammatory response to exercise (Peake et al., 2005; Chazaud, 2016). Based on the combination of measured parameters, particularly CRP, we cannot conclude that significant change in systemic inflammatory status was visible.

In conclusion, our study showed shift toward less inflammatory IgG N-glycosylation profile following intense RST that manifested as decrease in agalactosylated and increase in digalactosylated and monosialylated N-glycans. Limit of the study was a relatively small sample size, but the fact that it was a longitudinal intervention study, compensates for this shortcoming to some extent. Very little research was done in terms of exercise induced changes in glycosylation, so this work presents a source of novel information, while providing foundation for future research. IgG glycosylation was sensitive enough to detect anti-inflammatory effect of intense exercise on young and healthy individuals, despite it not being clearly visible using traditional inflammation markers, suggesting its potential as promising marker for long term pro/anti-inflammatory effects of physical exercise. This finding, of course, should be confirmed on larger sample size and different exercise paradigms.

## Data Availability Statement

The datasets generated for this study are available on request to the corresponding authors.

## Ethics Statement

The studies involving human participants were reviewed and approved by the Ethical Committee of the University of Zagreb, Faculty of Pharmacy and Biochemistry. The patients/participants provided their written informed consent to participate in this study.

## Author Contributions

TK conceived and designed the study with DM and OG. OG and GL supervised the study. MT, DM, DB, DK, and TK participated in data acquisition, collection, and analysis. All authors participated in interpretation and approved the final version of the manuscript. MT and TK drafted the manuscript. OG, GL, DM, DB, and DK critically revised the manuscript for intellectual content.

## Conflict of Interest

GL is the founder and owner of Genos Ltd., a company that specializes in high-throughput glycomics and has several patents in this field. The remaining authors declare that the research was conducted in the absence of any commercial or financial relationships that could be construed as a potential conflict of interest.

## Footnotes

1 https://glycostore.org/
