# Supplementary material for: Intense Physical Exercise Induces an Anti-inflammatory Change in IgG N-Glycosylation Profile
Source: Front Physiol. 2019 Dec 20;10:1522. doi: 10.3389/fphys.2019.01522 (PMC6933519; doi:10.3389/fphys.2019.01522)
Supplement: Supplementary file 1 [file Table_1.docx]

Supplementary Material

*Experimental protocol*

The complete experimental protocol was carried out within nine weeks. Anthropometric measurements were performed during the first and the final week of the experimental protocol along with the other fitness tests which were required as this study was conducted as part of a larger sports physiology study. In order to familiarize the participants with the training program two 60-minutes familiarizing training sessions were scheduled during the second week, for all participants before the formation of experimental and control groups. The training intervention started on the third week and lasted for six weeks during which 18 training sessions were performed. Training sessions were scheduled on the same time of the day (from 4 to 6 pm), three times a week, with a 48-hour lag between the sessions. All training sessions were performed in a handball-court gym on a wooden carpet floor and were supervised by a strength and conditioning coach. Blood samples were taken after: second familiarizing training, most intense training intervention (Week 6, Wednesday) and one month following the last training session.

Each training session started with the standardized warm up consisting of low-intensity running, dynamic stretching and progressive short-distance accelerations for total duration of 18 minutes. Main part of the session consisted of 2-3 sets of 6-10 straight-line or shuttle 20-meter repeated sprints with departures every 25 seconds and 2-minute inter-set passive recovery (Supplementary Table S1). Recovery between sprints was active as participants were required to jog back to the starting line and assume starting positions three seconds before the audio signal for the next sprint. The entire training protocol for each session was prerecorded as an audio file and was then played on a personal computer, so that timing for commencement of each sprint and duration of recovery interval could be fully controlled. After the start signal the participants were required to run the 20-meter distance as fast and possible. In order to comply with the progression and undulation principle of training the number of sets and repetitions was progressively increased throughout the six-week intervention period. During the training period the participants executed 338 sprints and covered 6 760 meters in total. In order to maintain maximal intensity throughout the entire training session constant verbal encouragement was provided by the training supervisor.

Supplementary Table S1 Experimental training program

|  | **Week 1** | **Week 2** | **Week 3** | **Week 4** | **Week 5** | **Week 6** |
| --- | --- | --- | --- | --- | --- | --- |
| **Monday** | 2 × 6 × 20 m  12 sprints | 3 × 6 × 20 m  18 sprints | 2 × 8 × 20 m  16 sprints | 3 × 8 × 20 m  24 sprints | 2 × 10 × 20 m  20 sprints | 3 × 10 × 20 m  30 sprints |
| **Wednesday** | 2 × 6 × 20 m  12 sprints | 3 × 6 × 20 m  18 sprints | 2 × 8 × 20 m  16 sprints | 3 × 8 × 20 m  24 sprints | 2 × 10 × 20 m  20 sprints | 3 × 10 × 20 m  30 sprints |
| **Friday** | 2 × 6 × 20 m  12 sprints | 3 × 6 × 20 m  18 sprints | 2 × 6 × 20 m  12 sprints | 3 × 8 × 20 m  24 sprints | 2 × 10 × 20 m  20 sprints | 2 × 6 × 20 m  12 sprints |
| **Total weekly training load** | 36 sprints  720 m | 54 sprints  1080 m | 44 sprints  880 m | 72 sprints  1440 m | 60 sprints  1200 m | 72 sprints  1440 m |

*Blood sampling*

Venous blood samples (2 mL) were collected within 20 minutes post-exercise, simultaneously at each experimental phase. Phlebotomy was performed in a sitting position after at least 15 minutes of rest by the same skilled phlebotomist according to the national recommendations for venous blood sampling by the Croatian Society of Medical Biochemistry and Laboratory Medicine (Nikolac *et al.*, 2013). The blood was collected in vacuum tubes containing K_2_EDTA (BD, Vacutainer) with 20-G straight needle venipuncture (BD) from the antecubital vein. All blood samples were then transported by car, stored in a cooled transport box, to the Endocrinology Laboratory of University Hospital Center Sestre Milosrdnice. Upon arrival at the laboratory, EDTA tubes were immediately centrifuged (at 1370 x g for 10 minutes) to separate erythrocytes from plasma. Subsequently, plasma supernatant was aspirated into a series of 1 mL aliquots and stored at - 80 °C until analysis.

Supplementary table S2 Formulas showing composition of derived glycan traits in terms of summation of relative areas of individual glycan peaks for igG N-glycans (A) and total plasma protein N-glycans (B)

| **A** | Derived IgG N-glycan traits | | |  |
| --- | --- | --- | --- | --- |
| Structural feature | | Formula |  |  |
| agalactosylated | | G0 = | GP1+GP2+GP3+GP4+GP5+GP6 |  |
| monogalactosylated | | G1 = | GP7+GP8+GP9+GP10+GP11+GP16 |  |
| digalactosylated | | G2 = | GP12+GP13+GP14+GP15+GP17+GP18+GP19+GP20+GP21+GP22+GP23+GP24 |  |
| asialylated | | S0 = | GP1+GP2+GP3+GP4+GP5+GP6+GP7+GP8+GP9+GP10+GP11+GP12+GP13+GP14+GP15 |  |
| monosialylated | | S1 = | GP16+GP17+GP18+GP19 |  |
| disialylated | | S2 = | GP20+GP21+GP22+GP23+GP24 |  |
| bisecting | | Bisecting = | GP6+GP10+GP11+GP13+GP15+GP19+GP22+GP24 |  |
| core fucosylated | | CoreFuc = | GP1+GP4+GP6+GP8+GP9+GP10+GP11+GP14+GP15+GP16+GP18+GP19+GP23+GP24 |  |
| oligomannose | | OligoMann = | GP5 |  |
| **B** | Derived total plasma protein N-glycan traits | | | |
| Structural feature | | Formula |  | |
| low branching | | LoBranch = | GP1+GP2+GP3+GP4+GP5+GP6+GP7+GP8+GP9+GP10+GP11+GP12+GP13+GP14+GP15+GP16+GP17+GP18+GP19+GP20+GP21+GP22+GP23 | |
| high b ranching | | HiBranch = | GP24+GP25+GP26+GP27+GP28+GP29+GP30+GP31+GP32+GP33+GP34+GP35+GP36+GP37+GP38+GP39 | |
| agalactosylated | | G0 = | GP1+GP2+GP7+GP19 | |
| monogalactosylated | | G1 = | GP3+GP4+GP5+GP6+GP13 | |
| digalactosylated | | G2 = | GP8+GP9+GP10+GP11+GP12+GP14+GP15+GP16+GP17+GP18+GP20+GP21+GP22+GP23 | |
| trigalactosylated | | G3 = | GP24+GP25+GP26+GP27+GP28+GP29+GP30+GP31+GP32+GP33+GP34+GP35 | |
| tetragalactosylated | | G4 = | GP36+GP37+GP38+GP39 | |
| asialylated | | S0 = | GP1+GP2+GP3+GP4+GP5+GP6+GP7+GP8+GP9+GP10+GP11+GP19 | |
| monosialylated | | S1 = | GP12+GP13+GP14+GP15+GP16+GP17 | |
| disialylated | | S2 = | GP18+GP20+GP21+GP22+GP23+GP24+GP25+GP26+GP27 | |
| trisialylated | | S3 = | GP28+GP29+GP30+GP31+GP32+GP33+GP34+GP35+GP36 | |
| tetrasialylated | | S4 = | GP37+GP38+GP39 | |
| core fucosylated | | CoreFuc = | GP1+GP2+GP4+GP5+GP6+GP10+GP11+GP13+GP16+GP17+GP22+GP23+GP31+GP34+GP35 | |
| antenary fucosylated | | AntFuc = | GP27+GP33+GP35+GP39 | |


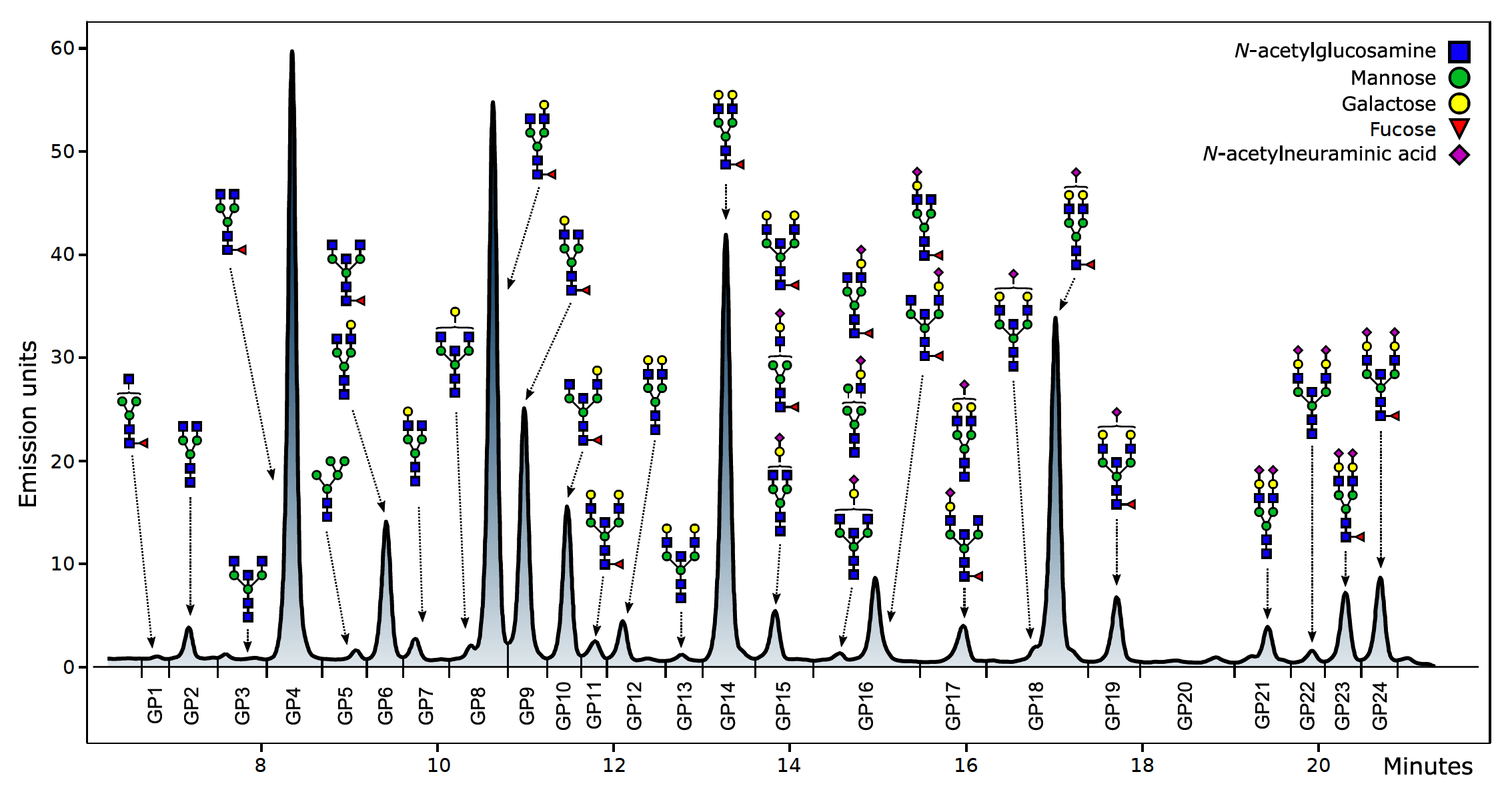


Supplementary figure S1 Example of IgG N-glycan chromatogram with the most abundant structures shown for each glycan peak


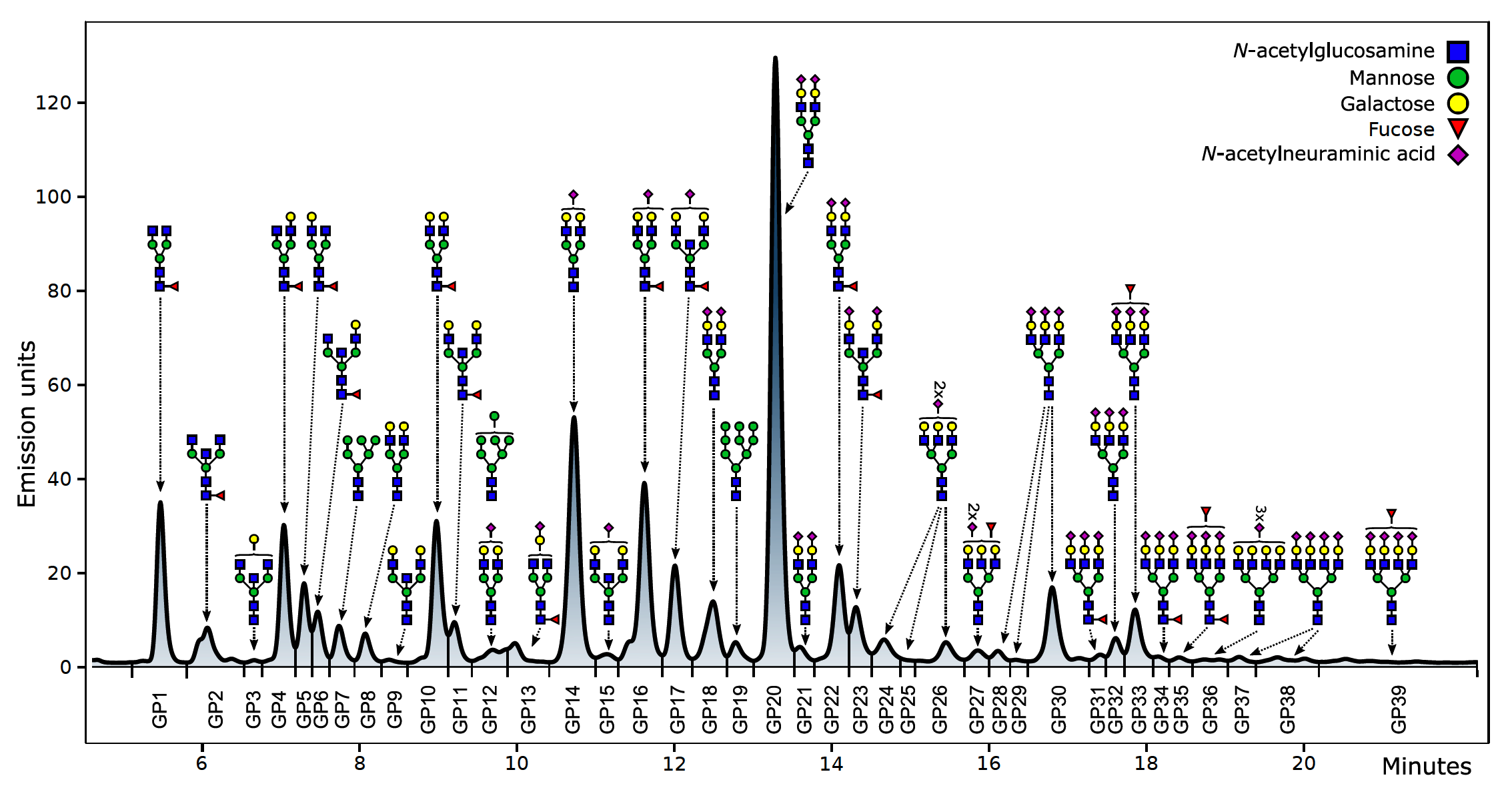


Supplementary figure S2 Example of total plasma protein N-glycan chromatogram with the most abundant structures shown for each glycan peak


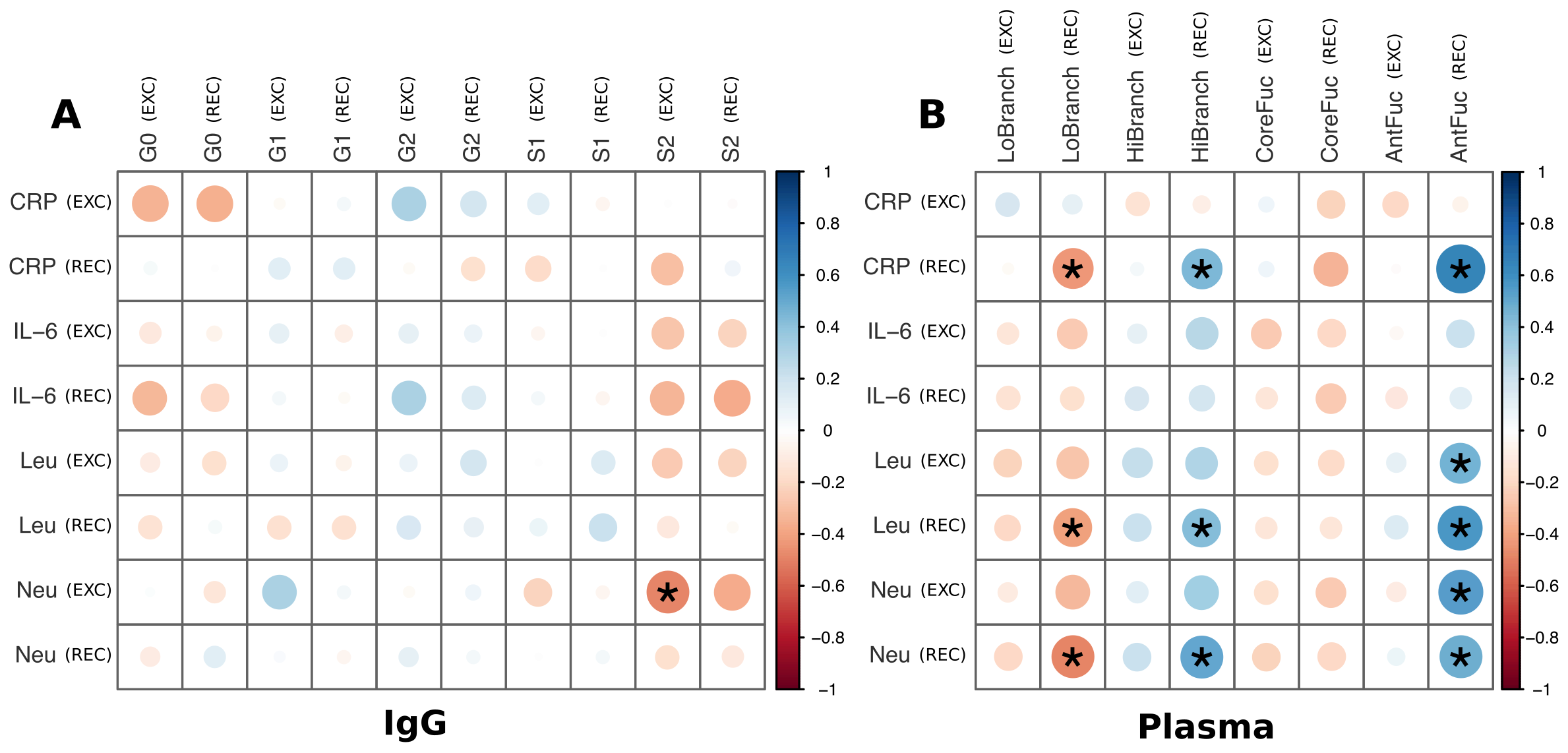


Supplementary figure S3 Correlation matrix showing calculated Kendall rank correlation coefficients between inflammatory marker levels and derived glycan traits for IgG (A) and plasma (B) for RST group. Asterisk (*) symbol represents correlations with unadjusted p-value lower than 0.05.

*References*

Nikolac, N. *et al.* (2013) ‘Croatian Society of Medical Biochemistry and Laboratory Medicine: national recommendations for venous blood sampling.’, *Biochemia medica*. Croatian Society for Medical Biochemistry and Laboratory Medicine, 23(3), pp. 242–54. doi: 10.11613/BM.2013.031.
